# Humidity as a non-pharmaceutical intervention for influenza A

**DOI:** 10.1371/journal.pone.0204337

**Published:** 2018-09-25

**Authors:** Jennifer M. Reiman, Biswadeep Das, Gregory M. Sindberg, Mark D. Urban, Madeleine E. M. Hammerlund, Han B. Lee, Katie M. Spring, Jamie Lyman-Gingerich, Alex R. Generous, Tyler H. Koep, Kevin Ewing, Phil Lilja, Felicity T. Enders, Stephen C. Ekker, W. Charles Huskins, Hind J. Fadel, Chris Pierret

**Affiliations:** 1 Center for Clinical and Translational Science, Mayo Clinic, Rochester, Minnesota, United States of America; 2 School of Biotechnology, KIIT University, Bhubaneswar, India; 3 Department of Biochemistry and Molecular Biology, Mayo Clinic, Rochester, Minnesota, United States of America; 4 Neurobiology of Disease Graduate Program, Mayo Clinic, Rochester, Minnesota, United States of America; 5 Department of Molecular Medicine, Mayo Clinic, Rochester, Minnesota, United States of America; 6 Department of Biology, University of Wisconsin- Eau Claire, Eau Claire, Wisconsin, United States of America; 7 Virology and Gene Therapy Graduate Program, Mayo Clinic, Rochester, Minnesota, United States of America; 8 Department of Biology Teaching and Learning, University of Minnesota, St. Paul, Minnesota, United States of America; 9 Aldrich Memorial Nursery School, Rochester, Minnesota, United States of America; 10 DriSteem, Eden Prairie, Minnesota, United States of America; 11 Department of Health Sciences Research, Mayo Clinic, Rochester, Minnesota, United States of America; 12 Department of Pediatric and Adolescent Medicine, Mayo Clinic, Rochester, Minnesota, United States of America; 13 Department of Infectious Disease, Mayo Clinic, Rochester, Minnesota, United States of America; Mount Sinai School of Medicine, UNITED STATES

## Abstract

Influenza is a global problem infecting 5–10% of adults and 20–30% of children annually. Non-pharmaceutical interventions (NPIs) are attractive approaches to complement vaccination in the prevention and reduction of influenza. Strong cyclical reduction of absolute humidity has been associated with influenza outbreaks in temperate climates. This study tested the hypothesis that raising absolute humidity above seasonal lows would impact influenza virus survival and transmission in a key source of influenza virus distribution, a community school. Air samples and objects handled by students (e.g. blocks and markers) were collected from preschool classrooms. All samples were processed and PCR used to determine the presence of influenza virus and its amount. Additionally samples were tested for their ability to infect cells in cultures. We observed a significant reduction (p < 0.05) in the total number of influenza A virus positive samples (air and fomite) and viral genome copies upon humidification as compared to control rooms. This suggests the future potential of artificial humidification as a possible strategy to control influenza outbreaks in temperate climates. There were 2.3 times as many ILI cases in the control rooms compared to the humidified rooms, and whether there is a causal relationship, and its direction between the number of cases and levels of influenza virus in the rooms is not known. Additional research is required, but this is the first prospective study suggesting that exogenous humidification could serve as a scalable NPI for influenza or other viral outbreaks.

## Introduction

Non-pharmaceutical interventions (NPIs) can complement traditional vaccination and anti-viral medications for infectious disease. NPIs are particularly significant for diseases like influenza, which have not shown to be well managed by vaccination alone. Worldwide, annual influenza epidemics are estimated to infect 5–10% of adults and 20–30% of children resulting in 3 to 5 million cases of severe illness and 250,000 to 500,000 deaths[1]. These infections account for 10% of global respiratory hospitalizations in children under 18 years of age[2]. Direct medical costs (2015) for influenza for inpatient care have been estimated at $3,650- $9,660 per case (reviewed in[3]) and a total economic burden of influenza exceeding $87 billion per year in the USA[4]. In Minnesota (MN), USA, the 2014–15 influenza season was especially severe with 4,202 people hospitalized with laboratory-confirmed influenza infection and 10 confirmed pediatric influenza-related deaths. This was the highest rate reported for the past six seasons including the H1N1 influenza pandemic in 2009[5]. Influenza and respiratory syncytial virus (RSV) accounted for over 50% of hospitalizations (all ages) from respiratory infections with an additional 706 outbreaks of influenza-like illness (ILI) reported in schools[5]. This increase in influenza was attributed to antigenic drift, leaving the vaccine largely ineffective. The 2014–15 influenza season illustrates why alternative approaches such as NPIs could prove to be valuable in disease prevention.

The probability for influenza virus transmission increases in situations where many people are in enclosed spaces and in close proximity such as airplanes and naval ships[6]. Children play a critical role in the transmission of acute respiratory infections within a community[7]. Survival and transmission of influenza virus may be impacted by the droplet size of airborne influenza. Larger droplets settle out of air at a more rapid rate, and do not penetrate as deep into the respiratory tract to seed infection[8]. Under laboratory conditions, others have shown that infectious influenza virus can persist from hours to days (17 days on banknotes with respiratory secretions[9]) in objects as varied as surface dust[10], cloth, pillow case, soft fabric toy, light switch material, formica, vinyl, and stainless steel[11, 12]. Researchers have demonstrated transfer of infectious influenza A virus from stainless steel surfaces (up to 24 hours) or from paper tissues (15 minutes) to hands, while remaining infectious on hands for 5[13] - 60[12] minutes.

The mortality and transmission rates of influenza A virus have been associated with decreased absolute humidity (AH) [14]. This epidemiological correlation suggests that deliberate increases in AH could be a potential NPI to reduce the spread of influenza and other viruses. One approach is to maintain relative humidity (RH) between 40–60%, the proposed optimal range for reducing growth opportunities for viruses, bacteria, and fungi[15]. Our previous study, demonstrated that classroom humidification to RH of 40–60% may be a feasible approach to increase indoor AH to levels with the potential to reduce influenza virus survival (a target of 10mb) and transmission as predicted by modeling analyses[16].

Community schools are promising locations for potential use of humidity as a NPI due to the role of children as a key source of influenza virus transmission from the community into a household[4]. We developed a novel in-school sampling process (S1 Fig) sensitive enough to detect influenza virus presence, quantity (viral RNA), and infectivity.

Outside of the laboratory, no prior studies have tested the potential for humidification to serve as an NPI. A few groups have succeeded in collecting influenza virus (RNA) from air samples in health care settings[17, 18]. One group also detected influenza virus (RNA) within air samples at a day-care facility (babies’ and toddlers’ rooms) and on board airplanes across the USA[18]. However, these studies did not detect and isolate infectious influenza from fomites in field conditions. This study investigated the presence and infectivity of influenza A virus in active preschool classrooms under control and humidified conditions.

## Results

**Fig 1:**
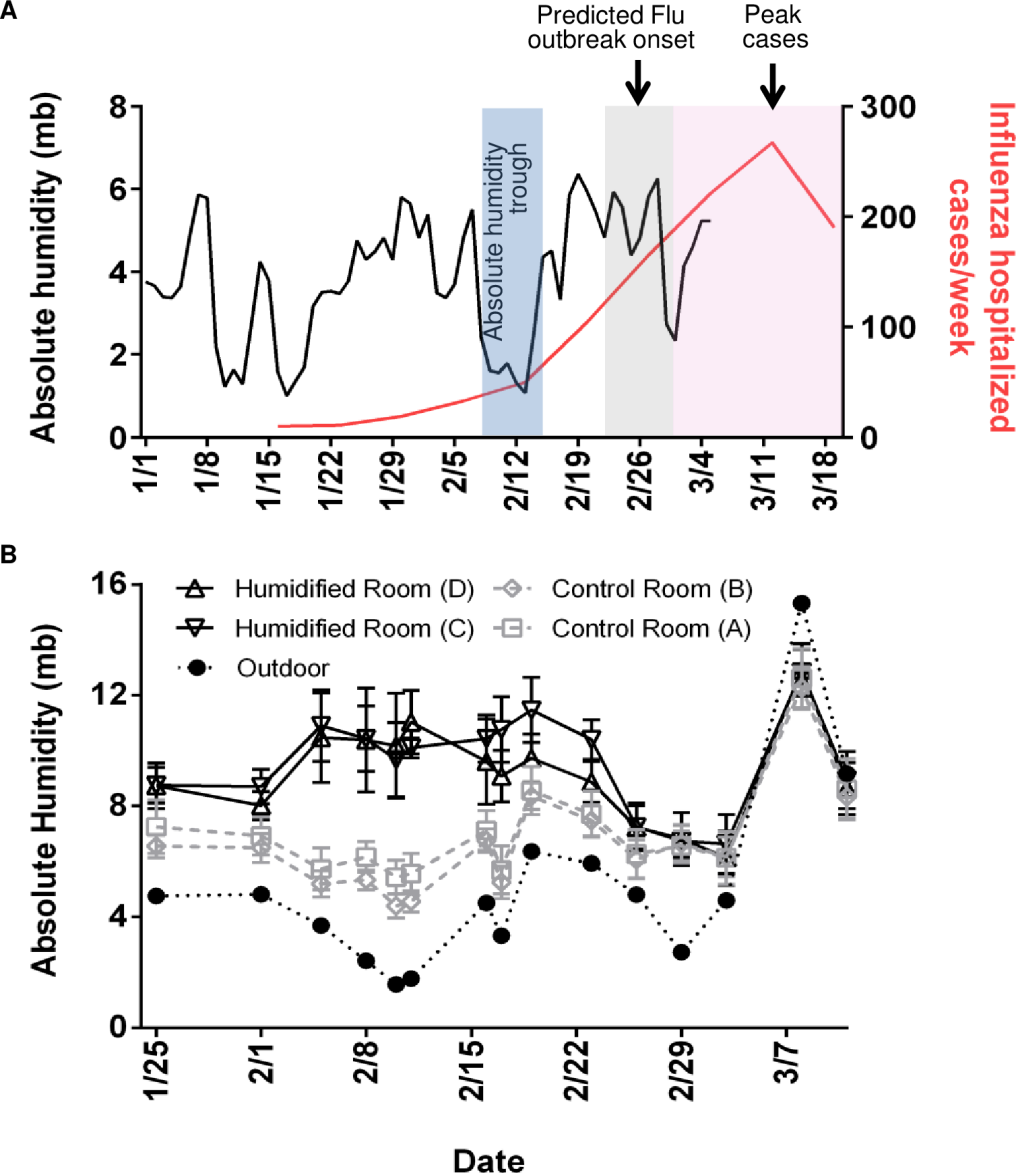
Absolute humidity and influenza hospitalized cases. (**A**) Outdoor absolute humidity (AH) values (n = 65; one measurement per day) from Rochester, MN and influenza hospitalized cases in MN (n = 1070, week ending in January 16^th^- March 19^th^). Applying the national trend model described by Shaman et al.[34] to the local humidity and illnesses, onset of influenza followed the predicted delay of 10–16 days (grey box) after an absolute humidity trough (blue box). Peak cases follow (pink box), as there is an incubation period of 1–4 days with viral shedding up to 7 days after symptoms resolve. (**B**) AH in 4 preschool classrooms (average of two sensors from 10 minute intervals over 150 minutes (n = 16 per sensor, room D) or (n = 17 per sensor, rooms A,B, C) per class period per sensor). Center values are mean of both sensors during class time and error bars are s.d. and corresponding outdoor AH (n = 15, 1 per day) on the 15 days of sample collection. Humidifiers were running in humidified rooms through sample collection on February 23.

At the end of the 2015–16 influenza season, an analysis of MN hospitalizations attributed to influenza virus (data supplied by MN Department of Health via FluSurv-NET)[19] revealed three troughs in atmospheric absolute humidity with the largest one (February 14, 2016)[20] preceding the peak of the seasonal influenza outbreak (Fig 1A). The peak for confirmed hospitalized influenza virus cases (confirmed positive) was the week ending March 12, 2016.

Elevated classroom humidification was maintained at an average of 9.89 mb in humidified rooms compared to 6.33 mb in control rooms (January 25 through February 23). AH was targeted near 10 mb based on previously demonstrated achievable levels in classrooms with a classroom humidifier running in winter [16]. We found that indoor school AH often reached 10 mb and 60% RH during late spring and early fall [16] and calculated 1-hour influenza virus survival of 35% (down from 75% of lowest measured AH of 2.67 mb). Student absences during the study period are presented in Table 1, followed by the samples positive for influenza A virus in Table 2.

**Table 1:** Student absences January 25 –March 11, 2016.

	Control		Humidified	
Category of Absence	% out	Total Attendance	% out	Total Attendance
General	9.17	1788	8.37	2293
Sick	1.29	1788	1.00	2293
ILI	0.39	1788	0.13	2293

Total attendance is the number of students enrolled in control or humidified classrooms multiplied by the total school days during sample collection. % out for general indicates students gone for all reasons (sickness, ILI, vacation) divided by total attendance. % out sick represents students who were not in class due to expressed illness divided by total attendance. % ILI out indicates that student had self-reported symptoms of fever plus cough or fever plus sore throat. Statistical analyses not run due to small sample sizes of absences.

**Table 2:** Influenza A positive samples by electrical impedance and RT-PCR assays from preschool.

		Control		Humidified		Control vs. humidified
SampleType	Assay	%positive	n	%positive	n	OR, [95% CI], P> |z|
Mixed (fomites and air)	Electrical impedance	48.1	27	16.7	18	LCS^**α**^
Mixed (fomites and air)	PCR	20	320	14.5	330	LCS^**α**^
Fomites	PCR	22.1	140	18.0	150	0.51, [0.33–0.78], 0.002
Air (total)	PCR	18.3	180	11.7	180	0.51, [0.29–0.89], 0.020
Air <1 μm	PCR	13.3	60	10.0	60	1.25, [0.91–1.72], 0.174
Air 1–4 μm	PCR	25.0	60	16.7	60	0.48, [0.16–1.42], 0.183
Air >4 μm	PCR	16.7	60	8.3	60	0.51, [0.23–1.10], 0.087

Percentage positive were calculated by dividing the number of positive samples by the number of samples taken in that condition. The sample number (n) is indicated. Samples tested by PCR included all fomites and air samples and are shown combined and separated into fomite and air (total) categories. Additionally air samples were separated into the three sizes of air particles collected. Statistical analyses of OR, 95% CI and P>|z| are indicated. **α** Indicates low cell size (LCS) so statistical analyses not run due to small sample size.

A total of 650 samples were collected (320 in control rooms, 330 in humidified rooms) of which 112 (17%) were positive for influenza A virus by RT-PCR (Table 2, S2 Fig). There were fewer influenza A virus positive samples in humidified rooms compared to control rooms for both fomites and for total air. However, when individual sizes of air particles were examined, differences did not achieve statistical significance. The distribution of influenza A virus positive samples within the different sizes of air particles varied with the greatest percentage of positive samples within the 1–4 μm size for both control and humidified rooms (Table 2).

**Fig 2:**
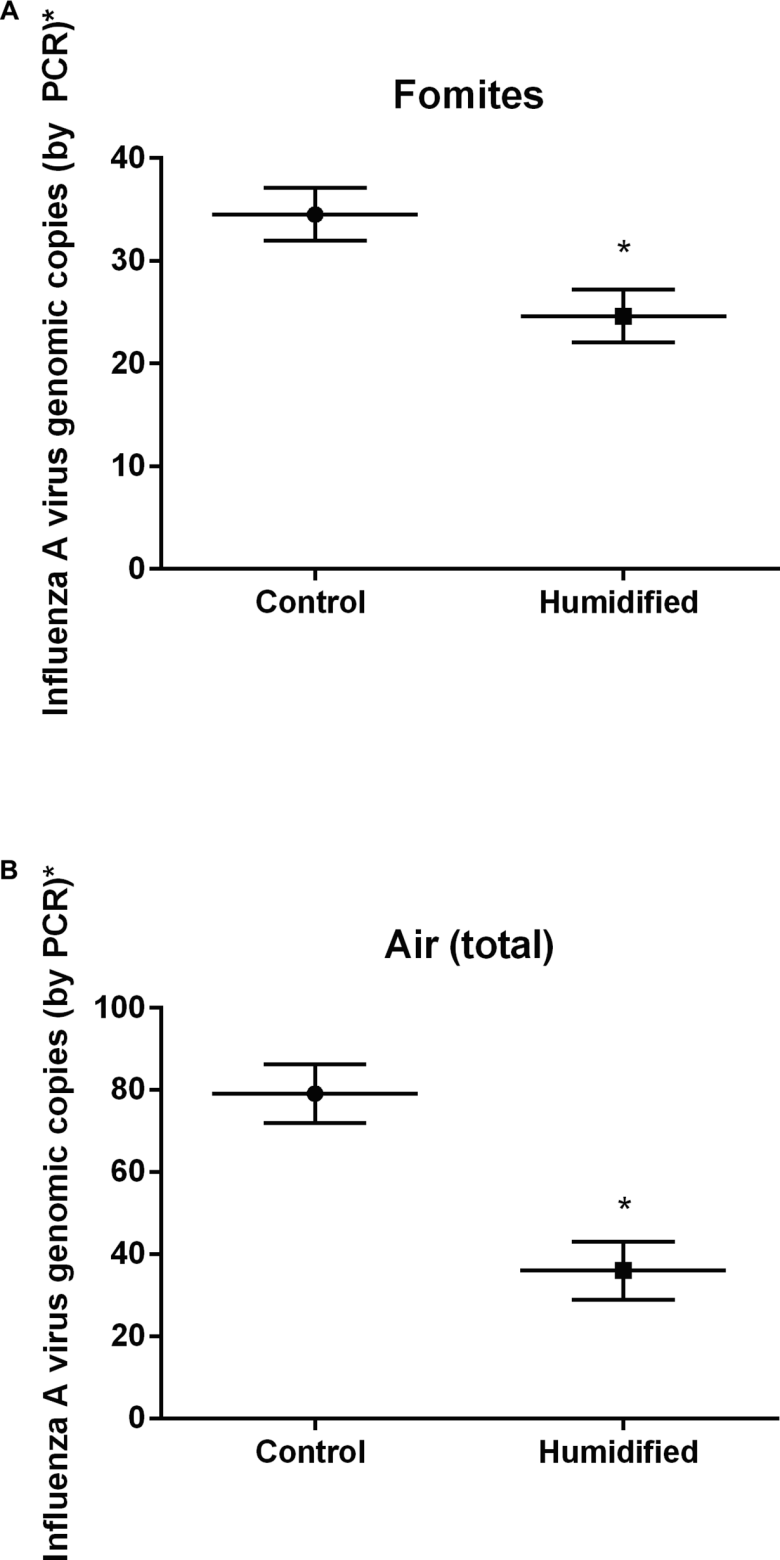
Influenza A virus genomic copies of positive samples. Horizontal bars indicate mean copy number and error bars are 95% CI. Fomites control, n = 31; Fomites humidified, n = 27; Air (total) control, n = 33; Air (total) humidified, n = 21. * Indicates air samples calculated mean per cubic meter of air based on air sample volume. * P<0.001.

Quantitative RT-PCR of influenza A virus for genome copy number revealed a significant reduction in mean copy number in humidified rooms compared to controls for fomites (P<0.001) and air (total) (P<0.001) (Fig 2A). The mean influenza A genomic copies per sample for humidified rooms was 24.6 compared to 34.5 for control rooms for fomites. For air (total) the mean influenza A genomic copies per cubic meter of air was 36.1 for humidified rooms compared to 79.1 for control rooms (Fig 2B). Review of qRT-PCR data at individual particle sizes revealed that air <1 μm (P = 0.010), and air > 4 μm (P = 0.011) experienced a significant reduction in influenza A virus mean genomic copies. This difference is displayed both as measured genomic copy number and as a calculated copy number per estimated air volume in S1 Table. Mean influenza A virus genome copies for air between 1–4 μm (P = 0.208) trended lower in humidified rooms but was not statistically different from control rooms at a 95% CI. Influenza A virus genome copies varied among different sample types with fomites having the highest copy number followed by air 1–4 μm particles (S1 Table). The electrical impedance assay has been used by other groups [21, 22] for assessing influenza A infectivity and for other viral infections [23, 24]. The instrument measures cell index as a read out of cell adherence, viability, and growth. When there are no cells present, the cell index is zero (as calibrated with wells containing plain media) and once the cells are seeded, attach and grow, the cell index increases. Electrical impedance assay revealed 35.6% of RT-PCR positive classroom samples tested to be infectious (Table 2 and S3 Fig). There were a smaller percentage of infectious samples from the humidified rooms (3 of 16 tested, 19%) than the control rooms (13 of 16 tested, 81%).

**Fig 3:**
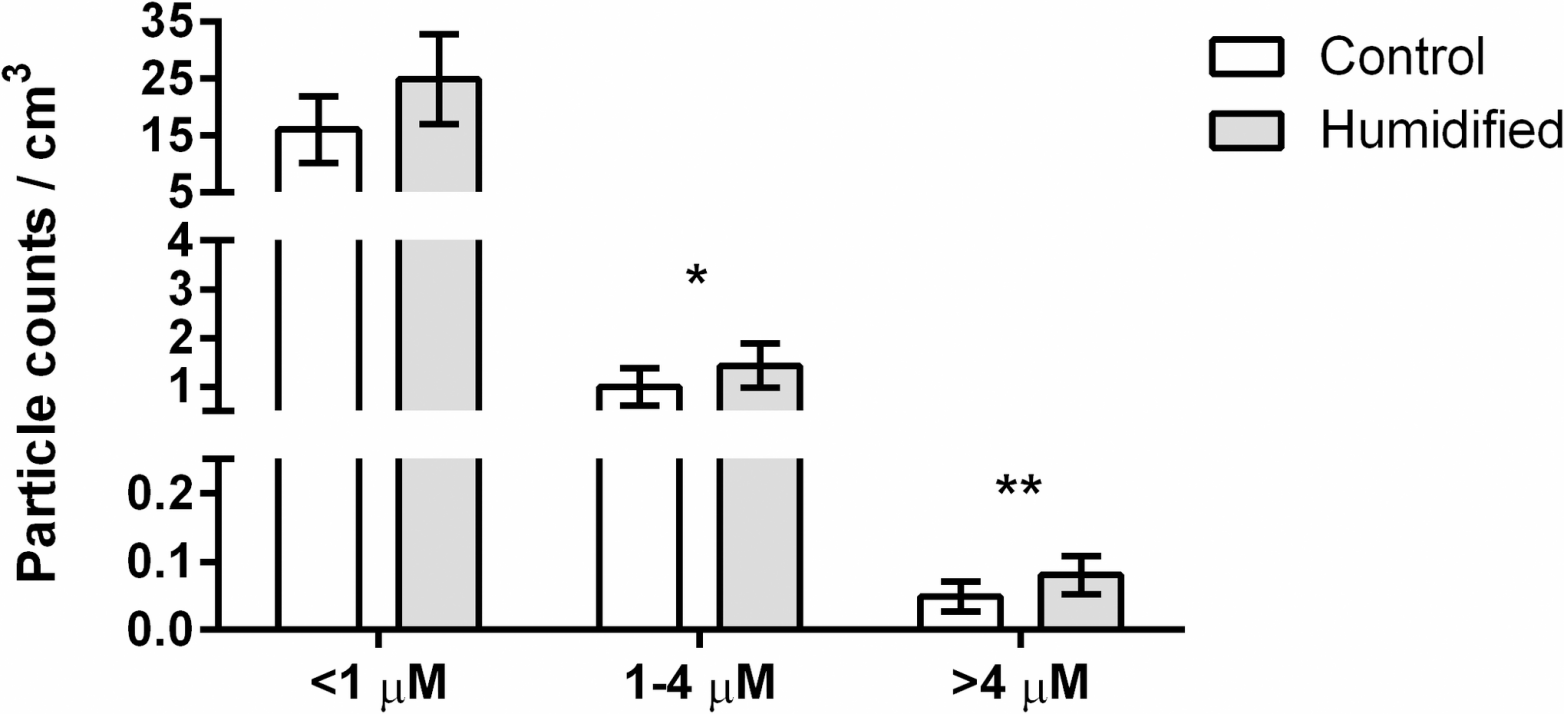
Air particle concentrations by size in classrooms. Bars indicate mean particle counts per cubic cm of air by size of air particles and error bars are 95% CI. Control, n = 23; Humidified, n = 30. * P <0.05, ** P <0.01.

The majority of air particles were less than 1μm size for both humidified and control rooms. Average particle concentrations (counts per cubic centimeter of air) decreased with increasing size in all four classrooms, irrespective of humidification status (Fig 3). Particles generated from the humidifier and the average counts from 7 independent measurements over a 22-minute period showed more than 96% of the particles provided by humidification were less than 1 μm. Humidified rooms showed a near doubling of both 1–4 μm, and >4 μm air particles (Fig 3). There were a significant increase in the population of larger sized particles (1–4 μm and >4 μm) in the humidified versus control rooms (Fig 3). This observation supports the hypothesis of droplet combination of the small particles added by humidification with present (and potentially influenza-bearing) particles. Larger particles remain airborne for less time (4 μm takes 33 minutes to settle 1 meter in still air versus 1 μm takes 8 hours)[17] and are unable to reach as deep within airways so are thought to be less pathogenic[8].

From January 25- March 11, 2016, 10 influenza-like illnesses (ILI) from absent students were recorded by school personnel (Table 1). Seven of these absences were from control (non-humidified) rooms and three were from humidified rooms. Both sets of rooms (control and humidified) had the same number of absences due to illness (23) overall. The differences in copy and infectivity are normalized to the number of positive samples. As shown in Table 2, in a per-room comparison of humidified and control rooms the % positive (# PCR+ /total samples per room), % infective (# positive by infectivity/ # PCR positive tested per room), and in Table 1 the % of students with ILI absences (# students with fever + cough or fever + sore throat / total student attendance by classroom during study) all were lower in humidified rooms than control rooms.

## Discussion

This study monitored presence, genomic copy number, and infectivity of influenza A virus in preschool classrooms during the dry winter months (low indoor humidity), which correspond with peak respiratory virus infections in Minnesota. An increase in average AH from 6.33 mb in control rooms to 9.89 mb in humidified rooms (RH ~42–45%) was associated with a significant decrease in influenza A virus presence in fomite and air samples in humidified rooms compared to control rooms. Additionally, PCR-positive samples from humidified rooms exhibited lower infectivity than samples from control rooms. It is important to note that this increase in influenza A viral genome copies and decrease in infectivity is shown normalized to the number of positive samples to eliminate the potential to interpret the increased copy number and infectivity as a simple expression of the number of sick children. The decrease in infectivity could be driven by a lower viral load of the PCR-positive humidified room samples as compared to PCR-positive control room samples. This correlates with the lower genome copies of influenza A virus PCR positive samples observed in humidified rooms. Enveloped viruses such as influenza are less stable in the environment than non-enveloped viruses and are more sensitive to higher relative humidity[25–27]. The exact mechanism underlying the action of humidity on the survival of the influenza virus is still not fully understood[28]. Several studies have hypothesized that surface inactivation for viruses with structural lipids may be due to denaturing of the lipoproteins found in enveloped viruses and phase changes in the phospholipid bilayer leading to cross-linking of associated proteins[25]. Additionally, relative humidity impacts the salt concentration within droplets and at low relative humidity (<50%), the solutes crystalize and influenza virus viability was maintained[29].

Prior studies have largely focused on the influence of humidity (RH or AH) on influenza virus survival under laboratory conditions ([8, 27–30]). One study modeled influenza virus survival across varied ranges of ambient indoor AH and humidification levels achievable in school environments[16]. Based on these findings, we hypothesized that classroom humidification might be a feasible approach to increase indoor AH to levels that could decrease influenza virus survival and transmission. Taking classroom humidification the next step further we included collection of samples from classroom environments and demonstrated that humidified rooms exhibited fewer influenza A virus-positive samples and reduced genomic copies. Additionally, influenza A virus-positive samples were less infectious in humidified rooms. Together, these outcomes strongly support the hypothesis that deliberate humidification can mitigate influenza A virus activity in a school environment.

In this study, humidifiers significantly increased the overall amount of particles, especially by increasing number of small air particles, leading to an increase in larger size particles, and rendering a shift in distribution towards largest particles, >4 μm. This could be attributed to the aggregation of particles in the humidified air, reducing the time of air suspension and transmission via inhalation (Fig 3). These findings enable a better understanding of the impact of humidity on influenza A virus survival (surfaces and objects) as well as virus transmissibility via aerosols by measuring the size of aerosol particles and the distribution of viruses within different sizes of particles.

Adding direct measurements of influenza virus (or other respiratory viruses) in a larger experimental population may reveal higher resolution outcomes to this approach. Furthermore, the impact of exogenous humidification on other viruses will need to be similarly measured to more fully understand the full potential impact of this NPI on other sources of communicable respiratory infections.

## Materials and methods

### Ethics statement

This study received prior IRB review from the Mayo Clinic Institutional Review Board and was approved as an IRB #15–000476, an exempt study (Not Human Subject Research). Consent from parents and guardians of the minors at Aldrich Memorial Nursery School was obtained for this study.

### Minnesota hospitalized influenza cases

Data supplied by the MN Department of Health regarding influenza hospitalizations for the 2015–2016 influenza season were fully anonymized prior to access by authors.

### Study site and absence data

The study was conducted at Aldrich Memorial Nursery School, Rochester, MN (a preschool with students aged 2–5 years) from January 2016-March 2016. Classrooms of identical design (see S4 Fig) each with their own HVAC system for air handling were utilized. Aldrich staff collected information on student absences (January 4, 2016- March 31, 2016) including ILI symptoms from students who were ill. ILI was defined as having fever plus cough or sore throat.

### Humidifiers, humidity measurements, and absolute humidity calculations

Model XTR (XTR003E1M) electrode steam humidifier with steam blower (SDU-003E) (DriSteem, Eden Prairie, MN) was installed in two experimental classrooms. The boiling of the softened tap water source provided decontamination of the steam distributed through the steam blowers. Classroom temperature and relative humidity were recorded every 10 minutes during the duration of the study with HOBO external data loggers, Model #U12-012 (Onset Computer Corporation, Bourne, MA). Two data loggers were installed per classroom, placed on the interior walls and set on top of the bulletin boards at a height of 2.032 meters. Data exported to Excel using HOBOware software (Onset Computer Corporation, Bourne, MA). Outdoor temperature and relative humidity was obtained from the North American Land Data Assimilation System (NLDAS) project[20]. Absolute humidity was calculated using Excel software using formulas as previously described[16].

### Bioaerosol sampler

NIOSH two-stage bioaerosol cyclone samplers[31, 32] collected air samples and separated them into three size fractions (>4 μm, 1–4 μm, and <1 μm) at a flow rate of 3.5 L / minute. NIOSH samplers were connected to AirChek XR5000 personal air sampling pumps, Model 224-PCXR4 (SKC Inc., Eighty Four, PA). Flow rate was calibrated using a Mass Flowmeter 4140 (TSI, Shoreview, MN), prior to each run of 150 minutes. Falcon conical tubes (15mL) (Corning, Corning, NY), 1.5 mL Fisherbrand microcentrifuge (Fisher Scientific, Pittsburgh, PA) and 37 mm hydrophobic Fluoropore PTFE membrane with a 3.0 μm pore size (EMD Millipore, Billerica, MA) were used to collect 3 different size fractions. Air sampling pumps were placed inside plastic ammunition boxes lined with mattress topper material (donated by Rest Assured Mattress Co, Rochester, MN). Cyclone samplers were affixed to the outside box surface with Industrial Strength Tape Strips (Velcro, Manchester, NH).

### Particle counts

After class dismissal, particle counts were measured in the center of each classroom at a height of 91 cm for 1 minute using a Six Channel Handheld Particle Counter, Model 23v750 (Grainger, ‎Lake Forest, IL). The particle counter had a flow rate of 2.832 liters per minute. Sizes of particles measured were 0.3, 0.5, 1, 2.5, 5, and 10 μm. Particle size data was binned into sizes to match those collected by the NIOSH samplers such that <1 μm included 0.3 and 0.5 μm sizes, 1–4 μm included 1 and 2.5 μm and >4μm included 5 and 10 μm. Particle counts were also measured before and after humidifier turned on in a pilot test. Particle counts were converted to counts per cubic centimeter by converting to cubic centimeters. 1L = 1000 cm^3^, so 2.832L x 1000 cm^3^,/ 1L = 2832 cm^3^. All particle count values were divided by 2832 to determine particle counts per cubic centimeter of air.

### Air samples

NIOSH samplers were dissembled in a BSLII biosafety cabinet. Collection tubes received 1 mL of infection media. Filters were retrieved from inside black polypropylene filter cassettes opened with a Stainless Steel SureSeal Cassette Opener (SKC Inc., Eighty Four, PA) and placed inside a 15 mL conical tube containing 1 mL infection media. The air filter was pushed down into the media using a sterile 1 mL serological pipette. Samples were placed on ice until ready for further processing.

### Fomites

25% cotton linen paper (Southworth, Neenah, WI) wrapped objects were provided to students. Objects included markers and a variety of wooden toys including blocks, rolling pins patterned wheel press and stamping cubes (Melissa & Doug, Wilton, CT). Additional items present in the classroom were also wrapped including rolling pins, hard rubber brayers, and plastic pizza cutters. After play, paper was transported back to BSL2 laboratory.

### Processing of fomites and air samples

Papers from classroom objects were lightly dusted with fingerprinting powder (Hi-Fi Volcano Latent Print Powder, Sirchie Youngsville, NC). Once fingerprint was identified, a portion of paper (~3.5–4 cm^2)^ was removed and placed into a 15 mL conical tube containing 1 mL of infection media. Samples were placed on ice until ready for further processing.

Both fomites (paper) and air samples (in media) were vortexed briefly and incubated on ice for 15 minutes. Samples were vortexed again prior to centrifugation for 20 minutes at 4°C at 3313g (15 mL tubes) and centrifuged at room temperature at 1520g in a microcentrifuge (1.5 mL tubes). Liquid was removed from 15 mL conical tubes and transferred to 1.5 mL conical tubes. The supernatant from these samples was frozen at -80°C and used for subsequent RNA isolation and qRT-PCR.

### Influenza A virus controls

Viral stocks of influenza A (H3N2) were provided by the Clinical Virology Laboratory, Mayo Clinic, Rochester, MN. Samples were thawed and used to infect bulk cultures of MDCK (NBL2) (ATCC CCL34) (ATCC, Manassas, VA). Influenza A virus infections followed published methods[33], except DMEM media was used instead of MEM. The contents of the flask were collected once cells became non-adherent and centrifuged 4°C at 3313g for 10 minutes to pellet cellular debris. Aliquots of supernatant were stored at -80°C.

### Influenza A virus infectivity assay

Samples were assayed for influenza A virus infectivity by electrical impedance assay[21] run on an xCELLigence RTCA MP instrument (ACEA Biosciences, Inc., San Diego, CA). The instrument measures cell index as a read out of cell adherence, viability, and growth. When there are no cells present, the cell index is zero (as calibrated with wells containing plain media) and once the cells are seeded, attach and grow, the cell index increases. After calibration with media, wells were seeded with MDCK cells. Twenty-four hours later, media was removed and the cells were washed with PBS. Wells were inoculated with serially diluted influenza A virus (positive controls), media (negative controls) and samples (tests). Readings were taken every 15 min for 7 days post inoculation. Using influenza A virus stock, dose dependent decline in cell index was demonstrated with 1:1000 dilutions, and gradual decline at 1:10000 and 1:100000 dilutions, whereas further dilutions did not impact cell index any differently than media controls (S3A Fig).

### Viral RNA isolation and detection using quantitative reverse transcription PCR (qRT-PCR)

The supernatant from fomite and air samples was thawed and 140 μL used for viral RNA isolation with the Viral RNA isolation kit (Qiagen) as per the manufacturer’s instructions, followed by RT-PCR analyses. The nonstructural (NS1 gene) sequence of influenza A virus was used for detection. See S2 Table for list of primers, product sizes and annealing temperature information. See S5 Fig for detection of viral RNA in multiplexed qRT-PCR. Briefly, SYBR green qRT-PCR was performed as described by the manufacturer (Qiagen SYBR green Quantitect kit). The PCR thermal profile consisted of an initial cDNA step of 30 minutes at 50°C followed by 15 minutes at 95°C and 30 cycles of 30 seconds at 95°C, 30 seconds at 56.5°C, and 30 seconds at 72°C. Detection, quantification and data analysis were performed in the CFX manager real-time detection system (Bio-Rad).

### In-vitro transcription of influenza A viral RNA

Though Influenza A and B and RSV were included in this analysis, low numbers of positive samples for RSV and Influenza B moved the focus of the study to influenza A virus only. Quantification of influenza A virus RNA was performed using in vitro transcription (IVT). The NS1 gene was amplified from a H3N2 influenza A virus isolate from infected MDCK cells using primers containing T7 promoter sequence in the forward sites: Inf AF: 5′-ACTGCTTAATACGACTCACTATAGGGAGATTTCACCGAGGAGGGAGCA -3′, Inf AR: 5′- CCTCCGATGAGGACCCCAA -3′;. The amplified NS1 gene was in-vitro transcribed with T7 RNA polymerase (Megascript T3 kit, Ambion, ABI) according to the manufacturer’s instructions for synthesizing short transcripts (105 bp) with the following modifications: incubation time increased to 8 hours and the enzyme mixture and template RNA was increased by three-fold of its original concentration. The synthesized RNA pellet was suspended in 0.1% diethylpyrocarbonate-treated water. RNA transcripts were purified, quantified and mixed with nuclease free water for preparation of positive controls in the range from 10^1^−10^7^ copies for the standard curve development. The amount of IVT-generated fragments was determined using the NanoDrop ND2000 Spectrophotometer (NanoDrop Technologies, Inc., Wilmington, DE) and converted to molecular copies according to the formula:

$$Y\frac{molecules}{\mu L}=\frac{\left(\frac{Xg}{\mu L}\;IVT\;RNA\;x\;6.02x10^{23}\right)}{\left(340\;x\;transcript\;length\;(bp)\right)}$$

The detection limit of the SYBR green real time RT-PCR assay was determined by testing serial ten-fold dilutions of the in vitro transcribed influenza A viral RNA ranging from 10^1^ to 10^7^ copies/μL. Cycle-threshold (Ct) values were plotted against the RNA copy number to construct the standard curve. The viral copy numbers of the processed samples were estimated by plotting the respective Ct values on the standard curve. See S6 Fig for standard curve example.

### Detection and quantification

Using serially diluted in vitro transcribed influenza A RNA, viral particles were detectable ranging from 10^1^–10^7^ RNA copies using the described assay. qRT-PCR sampling was sufficiently sensitive to detect as few as 7 RNA copies. To determine influenza A virus mean genomic copy per cubic meter of air we accounted for the volume of air based on air sampler collection at 3.5 L / minute for 150 minutes. 3.5 L / minute x 150 minutes x 1 cubic meter of air / 1000 L of air = 0.525 cubic meters of air collected per air sample. Mean copy number was divided by 0.525 to get influenza A virus mean genomic copy number per cubic meter of air.

### Statistical analyses

Percentage of samples positive for influenza A virus (Table 2): To account for within-room clustering for class cohorts, generalized estimating equations were utilized with a binomial family and logit link. Data are reported as odds ratio of positive test result for humidified rooms as compared to control rooms (each fomite or air sample accounting for a single positive or negative event), with a 95% confidence interval and p-value. Odds ratios below one (with statistical significance) demonstrate protective effects of humidification in that positive influenza virus was less likely to be obtained by the relevant capture system. Capture systems for influenza virus included paper and air (total, >4 μm, 1–4 μm, <1 μm); results were treated as positive for any capture within the room to minimize the effect of the different number of collectors per room for the paper and air capture systems.

Mean genomic copies of influenza A virus positive samples (S1 Table): To account for within-room clustering for class cohorts, generalized estimating equations were utilized with a gaussian family and identity link. Data are reported as the mean (standard deviation) of genome copy number for humidified rooms as compared to control rooms, with a 95% confidence interval and p-value. Capture systems for influenza virus included paper and air (total, >4 μm, 1–4 μm, <1 μm).

Mean particle count concentration data comparing humidified and control groups (Fig 3) for the three different sizes of particles (>4 μm, 1–4 μm, <1 μm) was analyzed in GraphPad Prism by unpaired t-test accounting for non-parametric data using Mann-Whitney test. P values were calculated from two-tailed analyses. Particle count data from time points where students were present in room for an additional enrichment activity were removed from analyses. Control (n = 23) and humidified (n = 30).

Additional statistical support was provided by The Biostatistics, Epidemiology and Research (BERD) Resource within the Mayo Clinic Center for Clinical and Translation Science (CCATS).

## Supporting information

S1 Fig Methodology flow chart of samples. (PDF)

S2 Fig Percentage of samples influenza A virus positive by day by qRT-PCR. The two lines represent humidity with average of control rooms (grey) and average of humidified rooms (black). The bars are the % of samples positive for influenza A virus (PCR). **(A)** Fomite samples (bars), n = 10 for control (grey) and humidified (black) rooms except for * where n = 5 for control. **(B)** Air samples (bars), n = 24 for control (grey) and humidified (black) rooms.
(TIFF)

S3 Fig Samples from preschool classrooms tested for infectivity by electrical impedance assay. Each line indicates a sample (well) for infectivity. Cell indices that returned to 0 indicated cell death (infectious). Arrows indicate individual samples. **(A)** Samples taken during humidification period (through February 23, 2016). MDCK cells were added at time 0 hours and media changed to samples (n = 19 in duplicate), influenza A virus positive control dilutions (6) in duplicate or control media (n = 11) at 24 hours. Additional controls in sample processing (media, paper, fingerprinted paper, hood control) were also included in duplicate as were 12 samples that were PCR negative. **(B)** Samples from February 26, 2016 and March 3, 2016 dates (post humidifier turn off).
(TIFF)

S4 Fig Floor plans of preschool classrooms including study classrooms. Four classrooms of identical size each with their own HVAC system for air handling were utilized. Control rooms (A & B) (non-humidified) and Humidified rooms (C&D) are indicated.
(PDF)

S5 Fig Detection of viral RNA using qRT-PCR. A indicates Influenza A virus. B indicates Influenza B virus. RSV indicates respiratory syncytial virus. Ta is annealing temperature, Tm is melting temperature and bp indicates size in base pairs.
(TIFF)

S6 Fig Quantification of influenza A virus positive samples with standard curve. **A)** Amplification curves of known copy numbers of NS1 gene of infuenza A. **B)** Melting curves of products from **A. C)** Standard curve showing standards (O) and experimental samples and positive controls (x) as labeled.
(TIFF)

S1 Table Influenza A virus mean genomic copies by qRT-PCR. Values indicate mean copy number for influenza A virus positive samples with 95% CI and P>|z| values. Statistics done on actual data collected (not based on volume of air). * Indicates air samples calculated mean per cubic meter of air based on air sampler collection volume. n.a. indicates not applicable as fomite samples were collected from pieces of paper, not air.
(TIFF)

S2 Table Sequences of primers used for real-time PCR. Allowed for simultaneous detection of influenza A virus, influenza B virus and RSV in one sample.
(PDF)
