# Supplementary figures and images for: Humidity as a non-pharmaceutical intervention for influenza A
Source: PLoS One. 2018 Sep 25;13(9):e0204337. doi: 10.1371/journal.pone.0204337 (PMC6155525; doi:10.1371/journal.pone.0204337)

**S1 Fig. Methodology flow chart of samples**

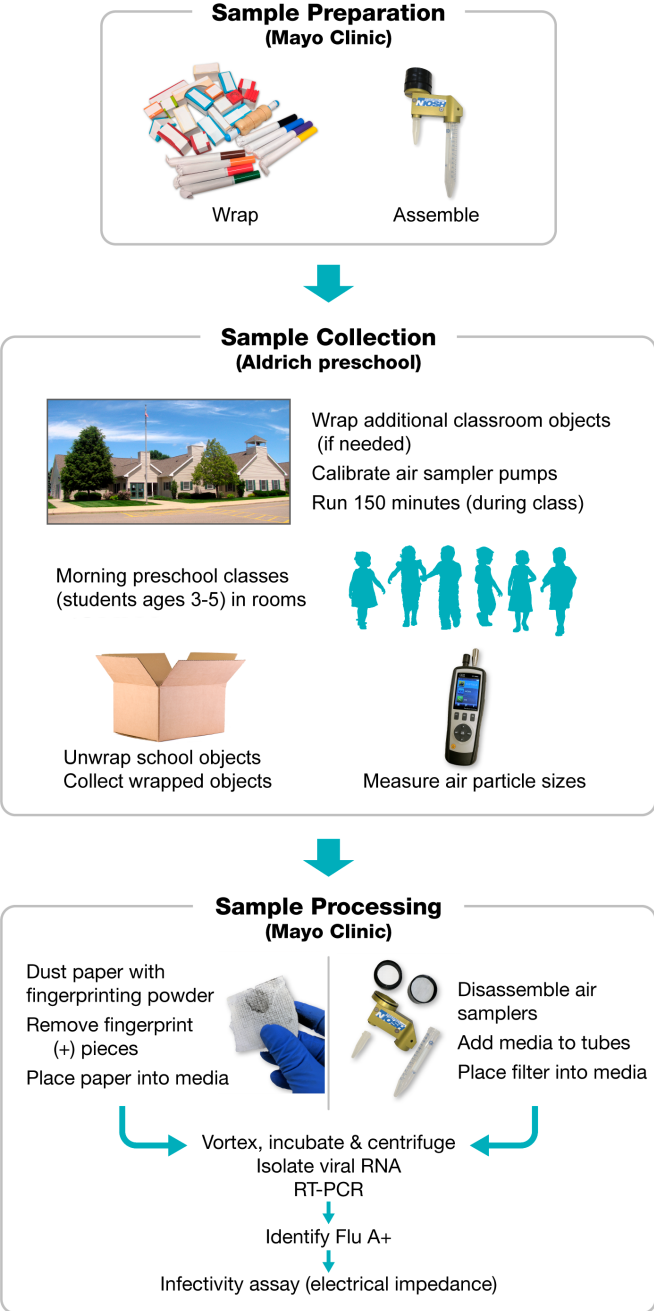

Supplement: S1 Fig — (PDF) [file pone.0204337.s001.pdf]

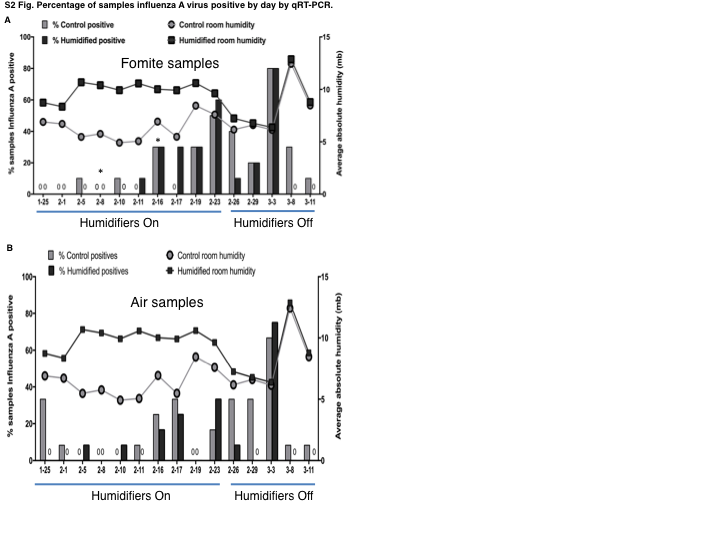

Supplement: S2 Fig — The two lines represent humidity with average of control rooms (grey) and average of humidified rooms (black). The bars are the % of samples positive for influenza A virus (PCR). (A) Fomite samples (bars), n = 10 for control (grey) and humidified (black) rooms except for * where n = 5 for control. (B) Air samples (bars), n = 24 for control (grey) and humidified (black) rooms. (TIFF) [file pone.0204337.s002.tiff]

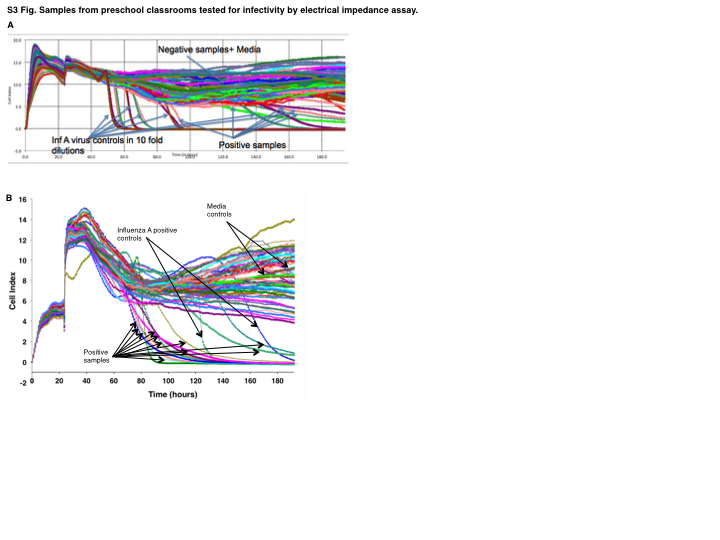

Supplement: S3 Fig — Each line indicates a sample (well) for infectivity. Cell indices that returned to 0 indicated cell death (infectious). Arrows indicate individual samples. (A) Samples taken during humidification period (through February 23, 2016). MDCK cells were added at time 0 hours and media changed to samples (n = 19 in duplicate), influenza A virus positive control dilutions (6) in duplicate or control media (n = 11) at 24 hours. Additional controls in sample processing (media, paper, fingerprinted paper, hood control) were also included in duplicate as were 12 samples that were PCR negative. (B) Samples from February 26, 2016 and March 3, 2016 dates (post humidifier turn off). (TIFF) [file pone.0204337.s003.tiff]

S4 Fig. Floor plans of preschool classrooms including study classrooms.

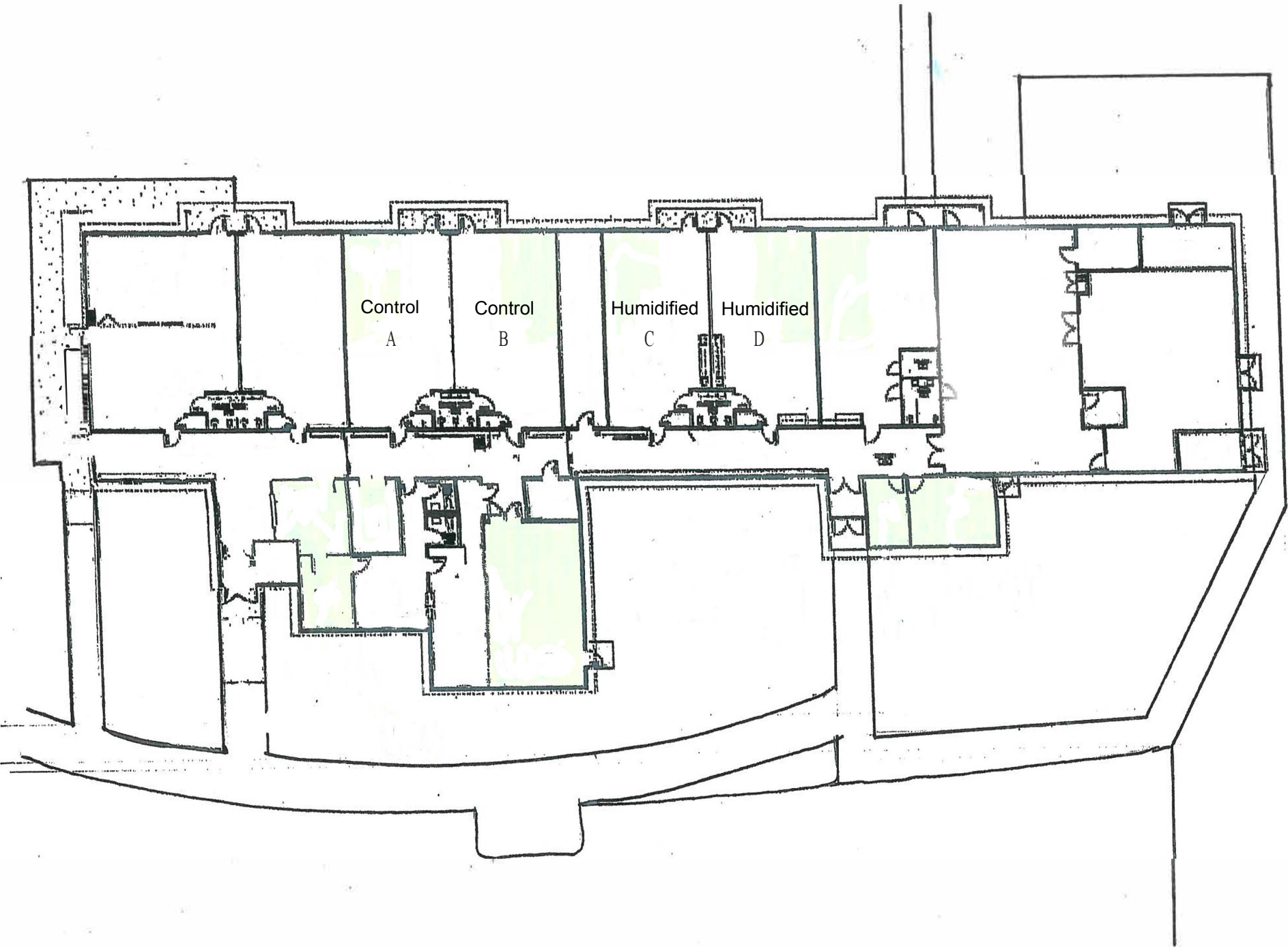

Supplement: S4 Fig — Four classrooms of identical size each with their own HVAC system for air handling were utilized. Control rooms (A & B) (non-humidified) and Humidified rooms (C&D) are indicated. (PDF) [file pone.0204337.s004.pdf]

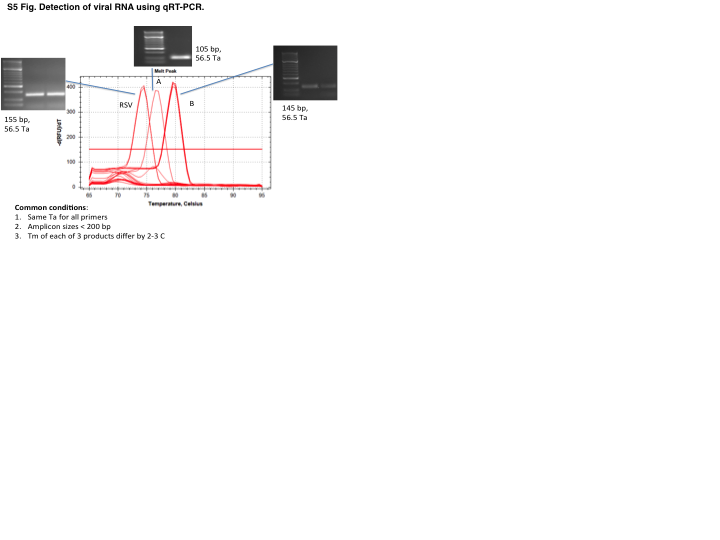

Supplement: S5 Fig — A indicates Influenza A virus. B indicates Influenza B virus. RSV indicates respiratory syncytial virus. Ta is annealing temperature, Tm is melting temperature and bp indicates size in base pairs. (TIFF) [file pone.0204337.s005.tiff]

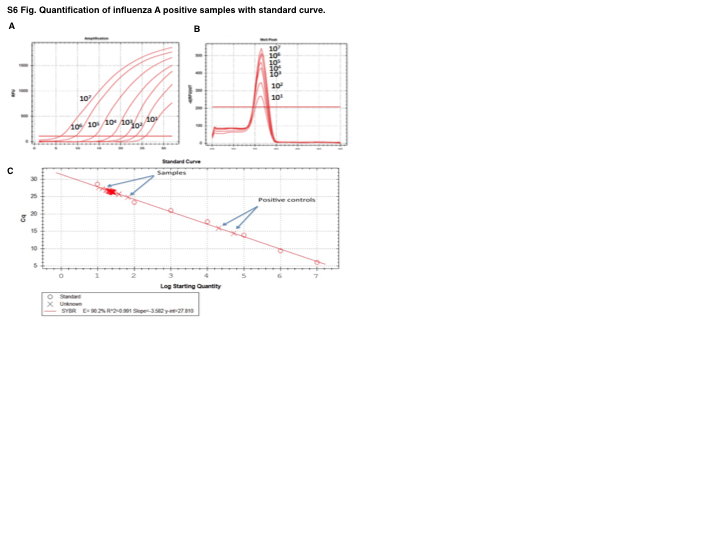

Supplement: S6 Fig — A) Amplification curves of known copy numbers of NS1 gene of infuenza A. B) Melting curves of products from A. C) Standard curve showing standards (O) and experimental samples and positive controls (x) as labeled. (TIFF) [file pone.0204337.s006.tiff]

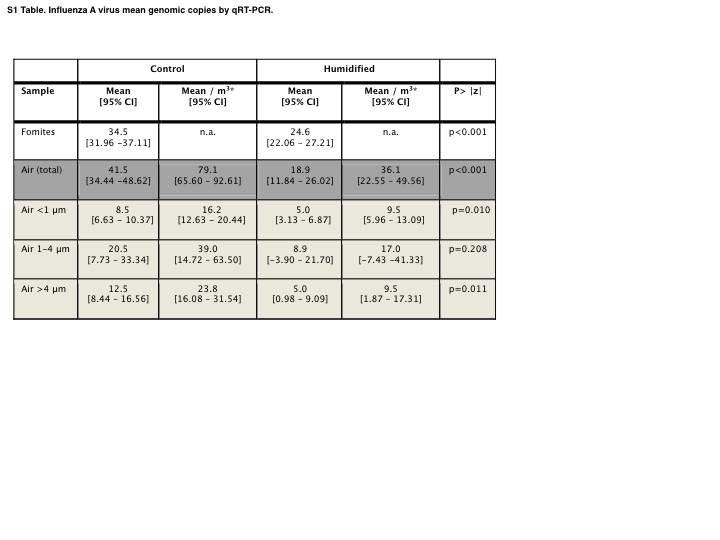

Supplement: S1 Table — Values indicate mean copy number for influenza A virus positive samples with 95% CI and P>|z| values. Statistics done on actual data collected (not based on volume of air). * Indicates air samples calculated mean per cubic meter of air based on air sampler collection volume. n.a. indicates not applicable as fomite samples were collected from pieces of paper, not air. (TIFF) [file pone.0204337.s007.tiff]
