# Supplementary material for: Humidity as a non-pharmaceutical intervention for influenza A
Source: PLoS One. 2018 Sep 25;13(9):e0204337. doi: 10.1371/journal.pone.0204337 (PMC6155525; doi:10.1371/journal.pone.0204337)
Supplement: S2 Table — Allowed for simultaneous detection of influenza A virus, influenza B virus and RSV in one sample. (PDF) [file pone.0204337.s008.pdf]

S2 Table. Sequences of primers used for real-time PCR.

Primer name	Primer Sequence	Product size (bp)	Annealing Temperature
InfA F	TTTCACCGAGGAGGGAGCA	105	56.5°C
InfA R	CCTCCGATGAGGACCCCAA		
Influenza Bs	GTCCATCAAGCTCCAGTTTT	145	
Influenza Bas	TCTTCTTACAGCTTGCTTGC		
RSVB F	GCATTAGCCAAAGCAGCAATAC	155	
RSVB R	CATCCATTAGACCATTTAATTGAGAGC		
